# Anisotropic thermal expansion of silicon monolayer in biphenylene network

**DOI:** 10.1039/d3ra06225b

**Published:** 2023-12-04

**Authors:** Aiqing Guo, Fengli Cao, Xiaodong Qiu, Weiwei Ju, Zhibin Gao, Gang Liu

**Affiliations:** a School of Physics and Engineering, Henan University of Science and Technology Luoyang 471023 People's Republic of China liugang8105@haust.edu.cn; b State Key Laboratory for Mechanical Behavior of Materials, Xi'an Jiaotong University Xi'an 710049 People's Republic of China

## Abstract

Materials with a negative thermal expansion property are of great importance in the emerging family of two-dimensional materials. For example, mixing two materials with negative and positive coefficients of thermal expansion avoids volume changing with temperature. In this work, based on first-principles calculations and Grüneisen's theory, we investigated the thermal expansion properties of a silicon monolayer in biphenylene networks. Our results show that the thermal expansion is greatly negative and anisotropic, as the linear thermal expansion coefficient along the *a*-direction is significantly smaller than the one along the *b*-direction, even at high temperatures. At 300 K, the thermal expansion coefficients along the two lattice directions are −17.010 × 10^−6^ K^−1^ and −2.907 × 10^−6^ K^−1^, respectively. By analyzing the Grüneisen parameters and the elastic compliance, we obtained an understanding of the giant negative thermal expansion of the material. Rigid unit modes are also responsible for the negative thermal expansion behavior. Our work provides fundamental insights into the thermal expansion of silicon monolayer in biphenylene networks and should stimulate the further exploration of the possible thermoelectric and thermal management applications of the material.

## Abstract 2

Materials with a negative thermal expansion property are of great importance in the emerging family of two-dimensional materials.

## Graphical abstract

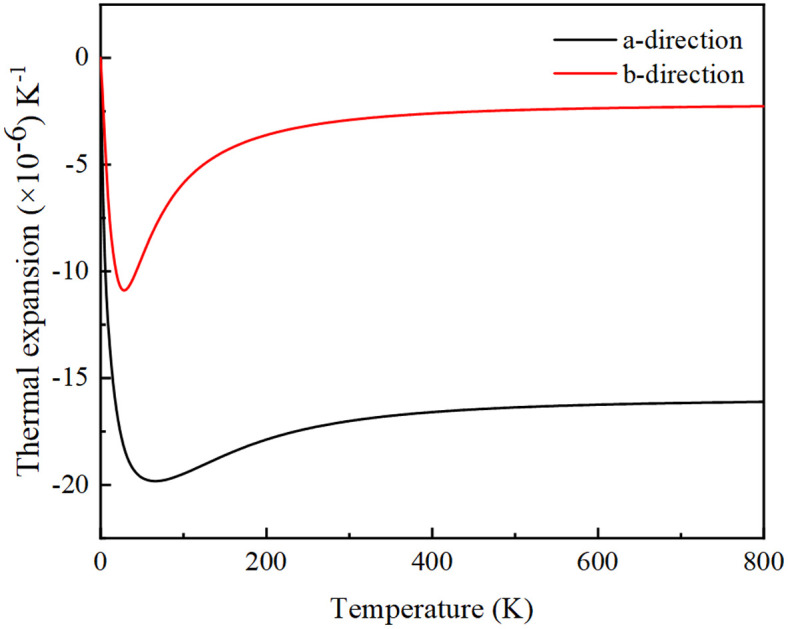

## Introduction

Since the successful exfoliation of graphene, two-dimensional (2D) materials have gained widespread attention and become one of the most active areas of nanomaterials research.^1–3^ Among them, the 2D group-IV materials in the same family as C are also developing rapidly due to their numerous remarkable physical properties and their great application potential.^4–8^ For example, the quantum Hall effect, valley polarization, tunable band gap and fast endowment mobility have been discovered in silicene and germanene with buckled hexagonal lattices.^1,9–15^ This indicates that they have great promise for applications in electronics, optics and fundamental physics research.

With the advancement of 2D materials research, quasi-one-dimensional (1D) carbon nanoribbons were synthesized in a biphenylene network on the surface of Au(111) by Fan *et al.*^16^ consisting of adjacent octagonal (o), hexagonal (h), and square (s) rings, called the ohs structure. It is a great achievement to realize a metallic, non-hexagonal, nanoscale monolayer of carbon proposed/predicted in various previous theoretical studies,^17–20^ paving the way for the exploration of new two-dimensional allotropes and their properties. The available studies show that the C ohs monolayer has excellent stability, electronic properties and thermal transport properties,^21,22^ and its thermal transport properties can be suppressed by hydrogenation.^23^ The C ohs monolayer is predicted to have an ultrahigh melting point up to 4500 K,^24^ negative thermal expansion (NTE)^25^ and a negative differential resistance effect.^26^ Moreover, C ohs monolayer is a good catalyst for oxygen reduction reaction,^27^ an effective gas sensor for NO and NO_2_,^26^ and a promising anode material for high-performance sodium-ion batteries.^28^ The superconducting critical temperature of the C ohs monolayer can be increased from 0.03 K to 11.22 K by adsorption of Li atoms, and the adsorption of alkaline metal atoms increases the transport anisotropy by more than two times.^29^ These findings suggest that C ohs have great potential applications, which prompted us to turn our research attention to the counterpart of other 2D group-IV materials in the same family.

As a cognate of carbon, the Si ohs monolayer has also attracted the attention of researchers. Salih *et al.* showed that the Si ohs monolayer is metallic and can be stacked to form vertical heterostructures, which can form metallic junctions and thus be applied to diodes.^30^ Tylan *et al.* showed that by dehydrogenation or deoxygenation stripes on Si ohs monolayers that are completely covered by H or O, various lateral composite structures can be constructed. And these structures consist of commensurately bare and atom-covered regions of ohs monolayers. Moreover, depending on the pattern and size of these regions, novel materials with unusual electronic and magnetic properties can be constructed, such as alloys, heterostructures, quantum wells, antipoints, and anticyclic rings.^31^

It is well known that the thermal expansion properties of materials are crucial. While the temperature changes, the accumulated thermal stresses and strains remarkably affect the performance and life of almost all devices. Mismatched thermal expansion of multiple materials may lead to severe device damage such as wire breakage and interface spalling. For example, Si is recognized as the next-generation anode material for lithium-ion batteries due to its extremely high theoretical capacity, low working potential and abundant natural abundance.^32^ Whereas, particle fragmentation due to volume expansion and crushing of Si particles are the major drawbacks to its commercialization.^32–34^ Therefore, pre-growing void space^35^ or adopting a finer particle structure^36^ is one of the ways to buffer volume expansion. However, it is even more crucial to select materials with appropriate expansion coefficients.

Although most materials exhibit positive thermal expansion when heated due to atomic vibrations,^37–40^ such as conventional single-crystal silicon with a linear coefficient of thermal expansion of about 2.6 × 10^6^ K^−1^ at room temperature,^41,42^ NTE has been reported for many 2D materials.^43,44^ For example, theoretical calculations predicted negative linear thermal expansion coefficients (TECs) for graphene, silicene, germanene, and phosphene,^45–47^ and several theories have been proposed to reveal possible physical mechanisms. The special temperature-responsive behavior of NTE, which violates the common sense of the “thermal expansion and cold contraction” effect, is of great scientific and technological significance. Some amount of NTE can facilitate the scaffolding of different materials into multicomponent structures, as NTE tends to counteract the usually harmful positive expansion and helps reduce thermal strain.^48^ Control of thermal expansion also expands the possibilities of designing nanoscale devices that can be expanded/contracted across a required temperature range. Therefore, materials with NTE properties are of great importance in the emerging family of 2D materials, and accurate measurement and prediction of TECs will also provide an important reference for the design of related functional devices. However, studies on the TECs of Si ohs monolayer structures are still lacking.

In this paper, we investigate the TECs of the Si ohs monolayer by first-principles calculations based on the Grüneisen theory, which can save a significant amount of computational time compared to routine quasi-harmonic approach (QHA), solving thermal expansion by the direct minimization of the total free energy. The results show that, similar to C ohs,^49^ the Si ohs monolayer contracts up to 800 K and has a higher thermal expansion anisotropy than C ohs. In addition, temperature-dependent lattice constants, generalized mode and macroscopic Grüneisen parameters, and the effect of elastic compliance tensor on TECs were investigated. It is found that the large and anisotropic NTE of the Si ohs monolayer over the entire temperature range is attributed to its large and anisotropic value of its macroscopic Grüneisen parameter (*G*). Rigid unit modes are also responsible for thermal expansion behavior. The revealed thermal expansion properties contribute to a further understanding of the study of thermal expansion and transport properties of 2D materials.

## Computational and theoretical methods

In this work, we used Grüneisen's theory^7,49^ to study the linear TECs of Si ohs monolayer. This method can save a lot of computational time, showing the efficiency and validity for anisotropic materials, compared to the routine QHA of direct minimization of the total free energy.^50–53^ Specifically, in the routine QHA method, a series of free energies calculations need to be performed on a grid of lattice parameter points, the dimension of which is determined by the number of independent lattice parameters.^4,54^ This implies the calculations of dozens or even hundreds of volumes of phonon spectra, requiring huge computational resources and time consumption. Moreover, it is well known that ZA modes are soft near the *Γ* point and their frequencies may become negative at larger strains. However, in order to accurately fit the energy data points to the equation of state, these data points should span a considerable wide energy range.^55^ This contradiction then affects the accuracy and validity of the method for two-dimensional anisotropic materials.^55^ Even with a much smaller computational cost, Grüneisen's theory can obtain good results of thermal expansion properties and has a wide range of applications.^43,51,56,57^ Therefore, we have investigated linear TECs for Si ohs monolayers using Grüneisen's theory.

The linear TECs of 2D materials with a rectangular lattice can be expressed as^49,57^:1
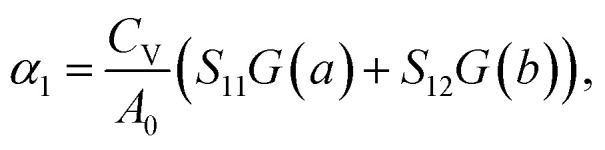
2
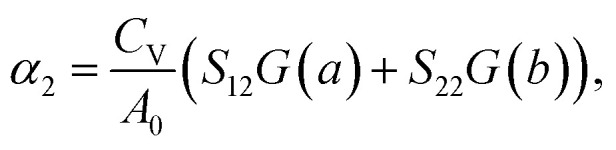
where *α*_1_ and *α*_2_ denote the linear TECs along the *a*- and *b*-directions, respectively. *C*_V_ is the constant volume specific heat. *A*_0_ is the area of the primitive cell and *S*_*ij*_ is the matrix element of the elastic compliance tensor, which is the inverse of the elastic stiffness tensor:3
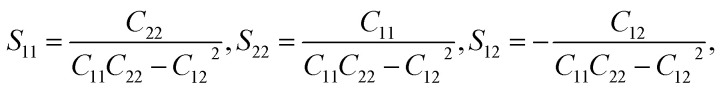
where *C*_*ij*_ is the matrix element of the elastic stiffness tensor. In addition, *G*(*a*) denotes the macroscopic Grüneisen parameter along *a*-direction, which is the average of the generalized mode Grüneisen parameter *γ*_*λ*_(*a*) along the same direction, weighted by *c*_*λ*_ (*c*_*λ*_ is the contribution of phonons with angular frequency *ω*_*λ*_ to the constant volume specific heat):4
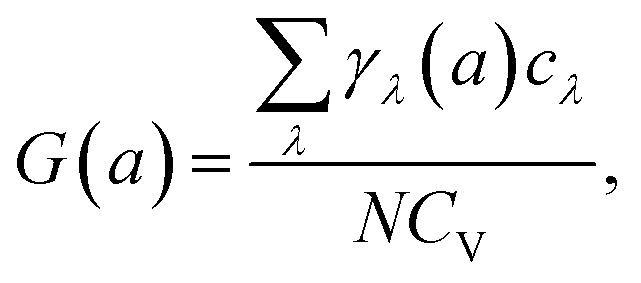
where *N* is the total number of wave vectors in Brillouin zone (BZ).

Moreover, the generalized mode Grüneisen parameter *γ*_*λ*_(*a*) can be calculated as:5
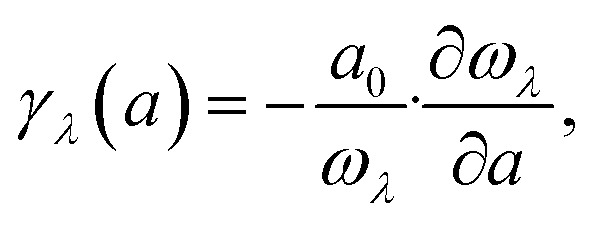
where *a*_0_ is the equilibrium lattice constant.

Our calculations were performed using the Vienna *Ab initio* Simulation Package (VASP)^58,59^ based on density generalized function theory. The projector-augmented-wave (PAW)^60,61^ pseudopotentials and Perdew–Burke–Ernzerhof (PBE)^62^ exchange–correlation functionals were chosen. Based on the existing structural model of the C ohs monolayer, the structural model of the Si ohs monolayer is determined by replacing C atoms with Si atoms. Unlike the planar structure of the C ohs monolayer, the Si ohs monolayer has a buckled structure. Therefore, a random perturbation was added to the *z*-axis of the lattice using VASPKIT^63^ before the structure optimization. To ensure the convergence, the plane-wave energy cutoff was set to 500 eV, much higher than the recommended value. A vacuum space of 18 Å was taken in the direction perpendicular to the crystal plane to prevent the interaction of two adjacent layers. In all calculations, the energy convergence was 10^−6^ eV and the maximal residual Hellmann–Feynman forces were reduced to 10^−4^ eV Å^−1^. And a 4 × 6 × 1 *k*-mesh was used during the structural relaxation. The phonon dispersions of Si ohs monolayer is obtained using the finite displacement method implemented in the PHONOPY code.^64^ To obtain the convergent phonon properties in the calculation of the harmonic interatomic force constants, a 5 × 5 × 1 supercell is used. After convergence tests, a 51 × 51 × 1 *q*-mesh is used for the calculations of harmonic properties.

## Results and discussions

**Fig. 1:**
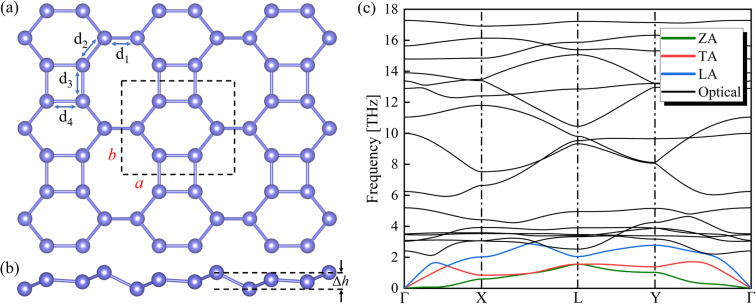
(a) Top view and (b) side view of Si ohs monolayer, where the rectangle represents the unit cell with *a* and *b* being the lattice constants. (c) Phonon dispersion of Si ohs monolayer. The coordinates of the high-symmetry points in the Brillouin zone are *Γ* (0,0,0), *X* (0.5,0,0), *L* (0.5,0.5,0), and *Y* (0,0.5,0).

The optimized Si ohs monolayer is shown in Fig. 1(a) and (b), consisting of octagons, hexagons and squares. The primitive cell of Si ohs structure contains six silicon atoms. The calculated equilibrium lattice constants are *a* = 7.13 Å, *b* = 5.73 Å, and the total buckling height is Δ*h* = 1.04 Å, which are in good agreement with the previous work.^30^ The Si–Si bond lengths are *d*_1_ = 2.323 Å, *d*_2_ = 2.285 Å, *d*_3_ = 2.292 Å and *d*_4_ = 2.287 Å as shown in Fig. 1. Based on the optimized structure, the phonon dispersion of Si ohs calculated using the PHONOPY code is shown in Fig. 1(c). As there are six silicon atoms in the primitive cell, eighteen phonon branches in the phonon spectra, including three phonon branches and fifteen photon branches. The absence of negative frequencies in the phonon spectrum indicates that the structure of the material is dynamically stable.

Essentially, the Grüneisen parameters are directly accountable for the TECs of the material. For 2D materials, the ZA mode is so soft that a slight change in the lattice constant may lead to a negative phonon frequency near the *Γ* point. This implies that the applied strain should be small enough. Therefor we expanded the lattice by 1% along the *a*- and *b*-directions, respectively, and used the same parameter settings to calculate the phonon dispersion of the Si ohs monolayer after stretching the lattice. Moreover, there are no negative frequencies in any of the three phonon spectra, ensuring the availability of the data.

**Fig. 2:**
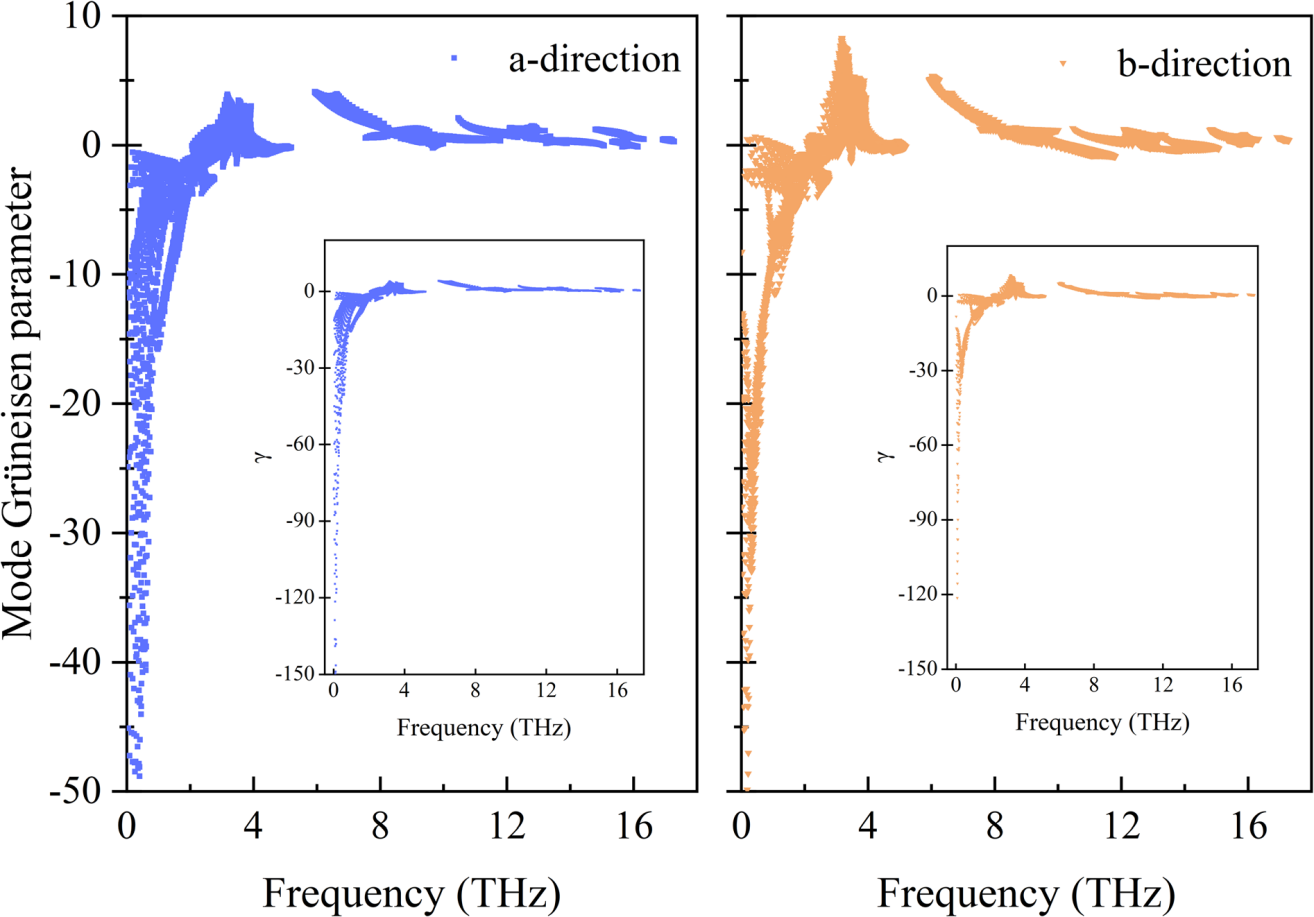
Frequency-dependent generalized mode Grüneisen parameters for the strain along the *a*- and *b*-directions for Si ohs monolayer. The insets show a large range of mode Grüneisen parameters along the corresponding directions.

The calculated frequency-dependent generalized mode Grüneisen parameters (*γ*) are shown in Fig. 2. The *γ* of the Si ohs monolayer shows a clear anisotropy. It can be seen from the figure that the overall trends of *γ* in both directions are very similar, but the distribution range of *γ* in the *a*-direction is much wider than that in the *b*-direction. Moreover, in the low frequency region, the *γ* values of phonons in the *a*-direction are much lower than those in the *b*-direction. And not only that, in the range of 3–6 THz, the *γ* values in the *a*-direction and *b*-direction also show obvious differences. Along the *b*-direction, it shows greater positive values of *γ*. In the high frequency region, *γ* changes from negative to positive values. Generally speaking, the negative value of *γ* indicates NTE. It means that the negative value of *γ* is the reason for the NTE of Si ohs monolayer, while its anisotropic thermal expansion is affected by the anisotropy of *γ*.

**Fig. 3:**
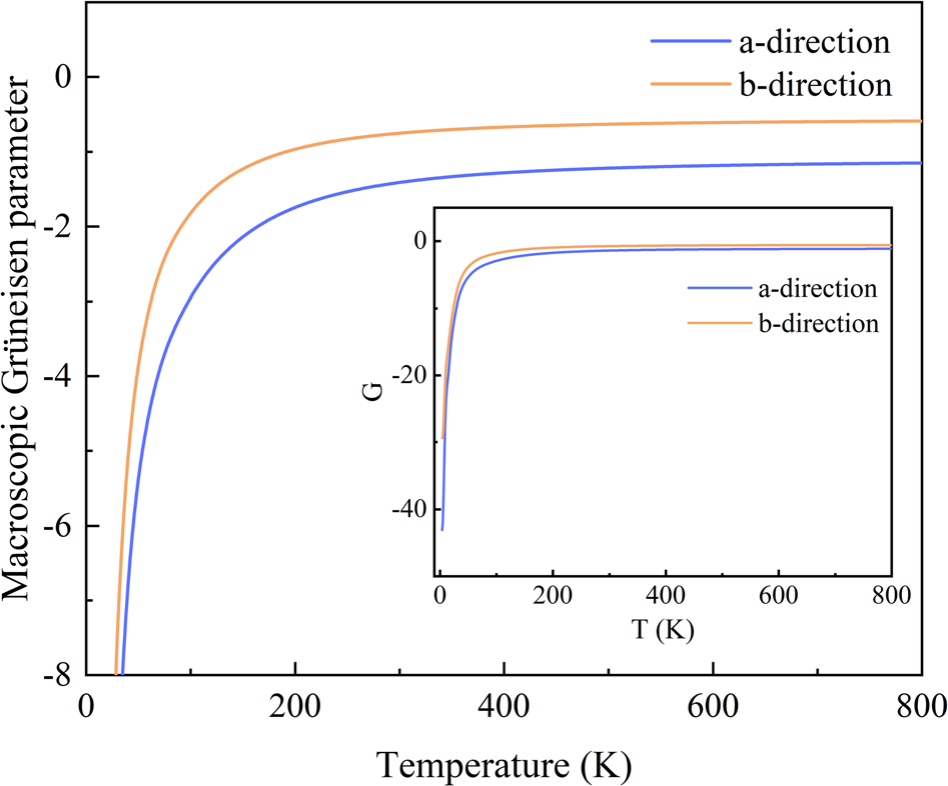
Variation of the macroscopic Grüneisen parameters with increasing temperature, along the *a*- and *b*-directions. The inset shows the results for the overall range.

The variation of *G* with temperature is shown in Fig. 3, and the inset shows the whole range of G. It is worth noting that the *G* are remarkably negative along both the *a*- and *b*-directions in the range 0–800 K. The reason is that a large number of phonons with negative *γ* are distributed over a rather wide range of frequencies, and the absolute values of them are large, up to 10^2^ orders of magnitude, while the positive values are below 10. At low temperatures, the predominantly excited phonon modes are low-frequency phonon modes with a large negative *γ*. As the temperature increases, high-frequency phonon modes, most of which have positive *γ*, are gradually excited. Thus, *G*(*a*) and *G*(*b*) increase rapidly with increasing temperature and reach their saturation values around 200 K. The saturation value of *G* along the *a*-direction is −1.161, which is lower than that of −0.594 along the *b*-direction. Moreover, in general, the range of *G* in the *a*-direction is much wider than that in the *b*-direction, consistent with the case of the *γ*.

**Fig. 4:**
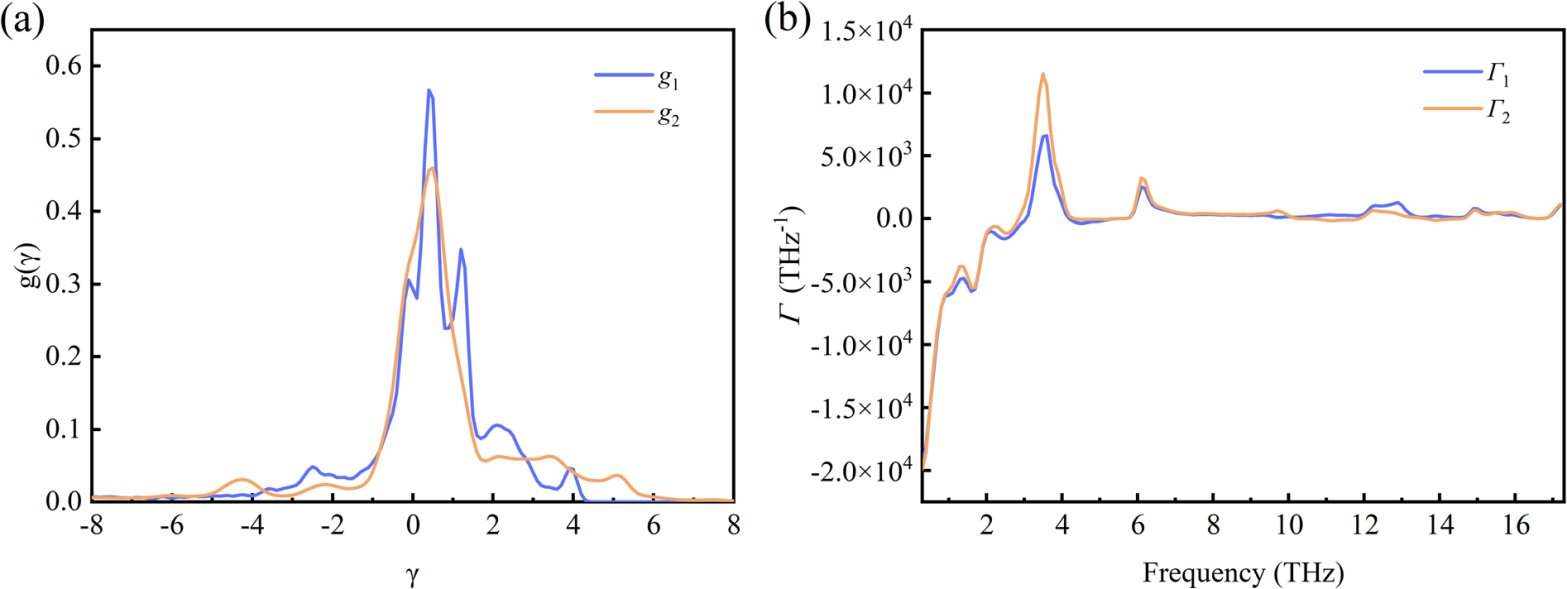
(a) The mode Grüneisen parameters density for Si ohs monolayer. (b) The frequency dependence of the mode Grüneisen parameters.

In order to clearly quantify the distribution of the Grüneisen parameters, we introduced the mode Grüneisen parameters density for uniaxial deformation along different directions.^50^ The density of the *γ* is expressed as 
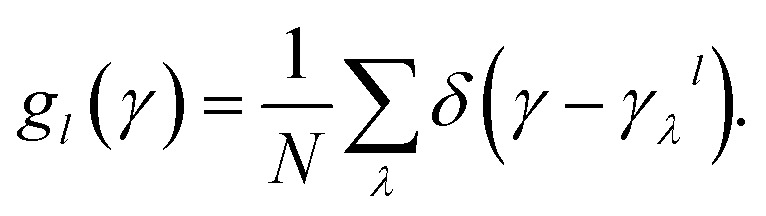
^50^ and displayed in Fig. 4(a). Note that *g*_1_ and *g*_2_ are the average densities of the *γ* along the *a*- and *b*-directions, respectively. As can be seen from the figure, the main distribution of *γ* ranges from −5 to 6. In the *a*-direction, the density reaches a maximum value of 0.57 when *γ* is 0.4, and *γ* reaches a maximum density value of 0.46 when *γ* is 0.5 in the *b*-direction. In addition, the percentages of phonons with positive *γ* values are more than 50% in both directions. In fact, we find that phonons with negative *γ* values occupy only 34.8% and 34.4% along the *a*-direction and *b*-direction, respectively. Combining with Fig. 2, we can notice that although the percentage of phonons with negative *γ* values is relatively low, the phonons with negative *γ* are dominant in thermal expansion.

To show the frequency-dependent information of the *γ*, we introduced *Γ*, the frequency-resolved phonon density of states weighted by the Grüneisen parameters, defined as 
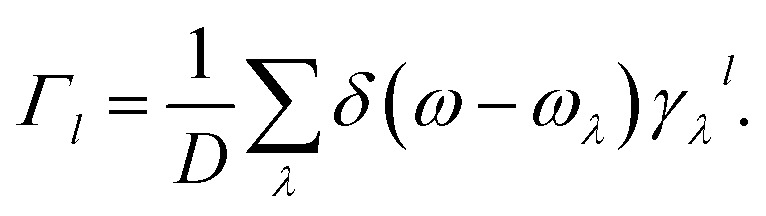
^50^ Here, *Γ*_1_ and *Γ*_2_ also correspond to the strains applied along the *a*- and *b*-directions. And *D* denotes the width of the interval in which the frequency is equally divided into multiple intervals. The frequency dependence information of the *Γ* is shown in Fig. 4(b). It can be seen that in the low frequency region (below 3 THz), the *Γ* values are all negative. Above 3 THz, the vast majority of *Γ* values are positive. It is also found the absolute value of negative *Γ* is larger than the one of positive *Γ* significantly, resulting in the negative values of the macroscopic Grüneisen parameters. And in general, the *Γ* values in the *b*-direction are generally larger than those in the *a*-direction, which is consistent with the same trend of the macroscopic Grüneisen parameters.

**Fig. 5:**
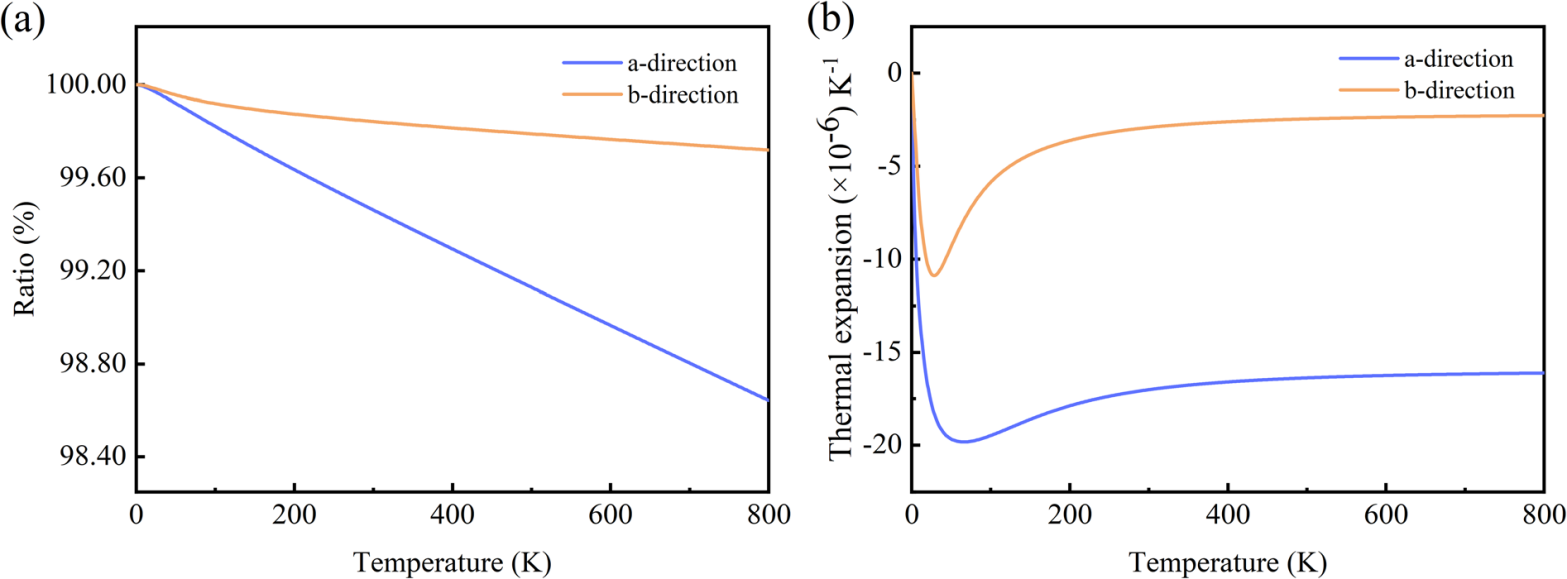
(a) The ratios of lattice constants for Si ohs monolayer. (b) Linear TECs for Si ohs monolayer.

In Fig. 5(a), we give the calculated ratios of the lattice constants *a* and *b* to the values of 0 K at different temperatures. As the temperature increases, both *a* and *b* are compressed, and *a* compressed much more than *b*. The ratios of the lattice constants are anisotropic like *γ* and *G*, and the anisotropy increases with the increase of temperature. From 0 to 800 K, it can be seen that the lattice parameters of the Si ohs monolayer shrink up to about 1.5 percent, which is greater than the 0.8 percent of C ohs^49^ and exhibits stronger anisotropy. And more precisely, at 300 K, the lattice constants *a* and *b* contract by 0.54% and 0.16%, respectively, and the percentage contraction increases to 1.36% and 0.28% at 800 K, respectively.

Fig. 5(b) shows the linear TECs with increasing temperature. As can be seen from the figure, the linear TECs of Si ohs monolayer decrease at first, then increases and finally stabilizes with increasing temperature. The linear TECs of many other 2D materials have the similar trend, such as graphene,^45,65,66^ hexagonal silicene,^45,47^ and C ohs monolayer.^49^ It is notable that the Si ohs monolayer exhibits giant NTE and anisotropy. At 300 K, the linear TECs reach −17.0 × 10^−6^ K^−1^ and −2.9 × 10^−6^ K^−1^ along *a*- and *b*-directions. For comparison, the linear TECs of C ohs are −11 × 10^−6^ K^−1^ and −9 × 10^−6^ K^−1^ along *a*- and *b*-directions.^49^ It is found the linear TECs of Si ohs monolayer are much lower in *a*-direction but higher in *b*-direction compared with C ohs monolayer. Thus, it shows more significant anisotropy. This significant in-plane anisotropy can be used for the thermal management of nanoelectronic devices and may attract more attention to orientation-dependent thermal devices.^67,68^

Here, we present the thermal expansion data of several related materials in Table 1. It is found that unlike the isotropic thermal contraction of graphene and silicene, the Si ohs monolayer and the C ohs monolayer are anisotropic. As shown in eqn (1) and (2), this is attributed to the anisotropy of their Grüneisen parameters, as the elastic compliance tensor *S* of Si and C ohs monolayers are nearly isotropic. The highest values of *G* for Si ohs monolayer in the *a*- and *b*-directions are −1.2 and −0.6 respectively, leading to the high anisotropy of the monolayer. And the absolute values of Grüneisen parameters for the Si ohs monolayer are much smaller than those of C ohs monolayer (−3 and −2.5 in the *a* and *b* directions, respectively). The much more significant anisotropy of *G* also results in more anisotropy of thermal expansion for Si ohs monolayer. Although the absolute values of *G* for the Si ohs monolayer are smaller, the *S*_*ij*_ are about 6.5–8.5 times larger than those of C ohs monolayer, finally leading to more remarkable NTE. Thus, it can be included that the elastic properties have an important effect on the thermal expansion, and soft materials may possess more remarkable positive/negative thermal expansion. Our calculations show that the *S*_11_ and *S*_22_ are positive, while *S*_12_ is negative. And the *S*_11_ and *S*_22_ are 2.38 and 2.47 times the absolute value of *S*_12_, respectively. The negative linear TECs are mainly determined by *G*. Both *G*(*a*) and *G*(*b*) are negative throughout the temperature region, which give rise to the negative linear TECs.

**Table 1:** Elastic compliance tensor and thermal expansion data for graphene, hexagonal silicene, C ohs monolayer, and Si ohs monolayer. Note that the superscripts of *a* and *b* here indicate the data along *a*-direction and *b*-direction, respectively

Material	Elastic constants (N m^−1^)		*S*_*ij*_ (m N^−1^)		*α* (×10^−6^ K^−1^)	Reference
Graphene	*C*_11_ = *C*_22_ = *C*_12_	312	—		−3.7	55 and 69
Hexagonal silicene	*C*_11_ = *C*_12_	68.9	*S*_11_	−0.0074	−5.3	55
	*C*_22_	23.3	*S*_22_ = *S*_12_	−0.0219		
C ohs monolayer	*C*_11_	283.2	*S*_11_	0.0040	−11(*a*), −9(*b*)	49
	*C*_22_	236.0	*S*_22_	0.0048		
	*C*_12_	88.5	*S*_12_	−0.0015		
Si ohs monolayer	*C*_11_	39.9	*S*_11_	0.0302	−17(*a*), −3(*b*)	This work
	*C*_22_	38.4	*S*_22_	0.0314		
	*C*_12_	16.1	*S*_12_	−0.0127		

**Fig. 6:**
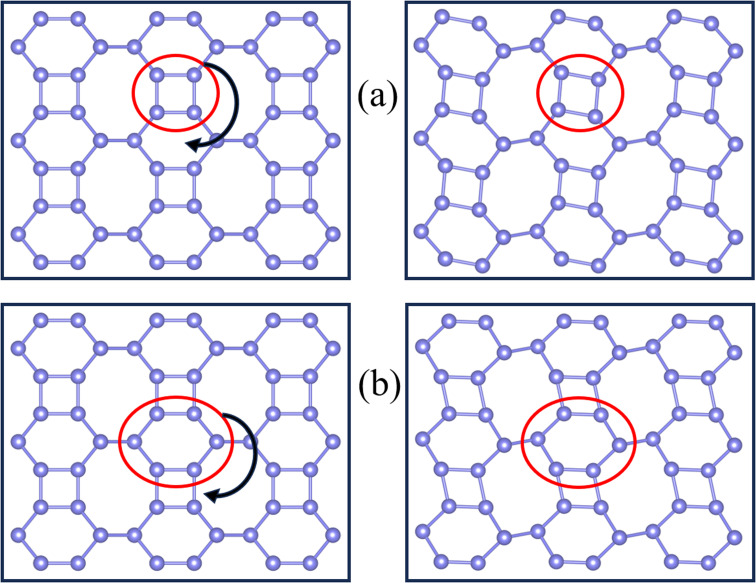
Real-space visualization of typical rigid unit modes observed in Si ohs monolayer with (a) *ω* = 3.09 THz, and (b) *ω* = 5.19 THz. Red circles indicate locally rigid units. The equilibrium structure is shown in the left box, while the right box depicts a snapshot of rigid units rotated clockwise.

In rigid unit mode theory, the NTE is also related to the vibrational modes of the rigid unit.^49,70^ So, we examined the real-space vibrations of all phonon modes at the *Γ* point. Fig. 6 shows the rigid cell modes (square and hexagonal rings) observed at *ω* = 3.09 THz and *ω* = 5.19 THz. We can find that the rigid unit modes are significantly rotated. During the rotation of rigid units such as the square/hexagonal ring in Fig. 4(a) and (b), other rings are deformed to fill the empty space, causing the strong shrinkage of the material. This phenomenon also occurs in C ohs monolayer.^49^

## Conclusions

In summary, we have investigated the thermal expansion properties of Si ohs monolayer through first-principles calculations. Our calculations show that the Si ohs monolayer exhibits giant NTE behavior in the temperature range of 0 to 800 K. Moreover, the absolute value of the linear TECs in the *a*-direction of the Si ohs monolayer is much larger than that of the C ohs monolayer over the entire temperature range, while the one in the *b*-direction is smaller than that of the C ohs monolayer. The linear TECs of Si ohs monolayer at 300 K are −17.0 × 10^−6^ K^−1^ and −2.9 × 10^−6^ K^−1^ along the *a*- and *b*-directions, respectively. The linear TECs along the *a*-direction is approximately 6 times greater than that along the *b*-direction. The in-plane thermal expansion exhibits a much stronger anisotropy than the C ohs monolayer. Our investigations suggest that the large negative value of the *G* and the rigid unit modes are responsible for its strong NTE, and the anisotropy of *G* mainly leads to anisotropic thermal expansion in the Si ohs monolayer. Furthermore, the elastic compliance is studied, confirming that soft materials usually possess significant thermal expansion. Our results on the NTE of Si ohs monolayer enrich the range of NTE 2D materials and will have a significant impact on the fundamental understanding and potential applications of Si ohs monolayer in electronic devices.

## Data availability

The data that support the findings of this study are available from the corresponding author upon reasonable request.

## Author contributions

Aiqing Guo contributed to the writing of one original draft. Gang Liu, Fengli Cao, and Xiaodong Qiu contributed to the writing, review, and editing of this article. Weiwei Ju and Zhibin Gao contributed to the conceptualization and supervision. All authors contributed to this article and approved the submitted version.

## Conflicts of interest

There are no conflicts to declare.

## Supplementary Material
